# Significance of circulating tumor cells detection in tumor diagnosis and monitoring

**DOI:** 10.1186/s12885-023-11696-3

**Published:** 2023-12-06

**Authors:** Yuanrui Liu, Rong Zhao, Zaichun Xie, Zhiyu Pang, Shengjie Chen, Qian Xu, Zhanfeng Zhang

**Affiliations:** 1 Department of Laboratory Medicine, Nanfang Hospital, Southern Medical University, Guangzhou, Guangdong 510515 China; 2 Clinical Laboratory, Guangzhou 8th People’s Hospital, Guangzhou Medical University, Guangzhou, Guangdong 510060 China; 3 Clinical Laboratory, The First Affiliated Hospital of Guangzhou University of Chinese Medicine, Guangzhou, Guangdong 510405 P.R. China

**Keywords:** Circulating Tumor cells, Tumor diagnosis, CTCs, Tumor early detection

## Abstract

To detect circulating tumor cells (CTCs) in the peripheral blood of patients with tumor, and to analyze the significance of CTC detection in tumor diagnosis and monitoring. In the present study, peripheral blood was collected from 125 patients with tumor, and CTCs were isolated and identified. Differences in CTC number and subtype detection were analyzed for different tumor diseases and stages. CTCs were detected in 122 of the 125 patients with tumor, with a positive rate of 97.6%. The number of CTCs increases in patients with vascular metastasis. The number of mesenchymal CTCs increases in patients with lymph node or vascular metastasis. The average ratio of epithelial CTCs in each positive sample decreases in the later stages of cancer compared with the earlier stages, while the average ratio of mesenchymal CTCs increases in the later stages of cancer compared with the earlier stages. The results showed that CTCs with mesenchymal phenotypes are closely related to lymph node or vascular metastasis. CTC detection can help with early diagnosis of tumor diseases. Continuous monitoring of changes in CTCs number and subtypes can assist clinical judgment of tumor disease development status and prognosis.

## Introduction

Tumors are often difficult to be cured and have a high mortality rate, making them a cause for concern. Currently, surgery and chemotherapy are the main treatment methods for early to mid-stage tumors, which can achieve certain results, including remission. However, tumor recurrence is often an unavoidable fact [1], so cancer patients often undergo regular check-ups [2, 3]. However, most check-up methods rely on imaging, which can only observe tumors after they have reached a certain size. This often results in missing the best treatment window. Early observation or monitoring of tumor development is undoubtedly the key to early detection and treatment of tumors [4].

CTCs are tumor cells that detach from primary or metastatic lesions and enter the bloodstream through the vascular and lymphatic systems [5]. CTCs can be thought of as the seed cells of malignant tumor metastasis. Several CTCs (at least three cells) can aggregate to form circulating tumor microemboli (CTM), which have a survival advantage over single cells because they can undergo epithelial-mesenchymal transition (EMT) to lose adhesion and become more easily metastasized while maintaining internal connections to resist apoptosis [6, 7].

CTCs detection is a novel liquid biopsy technology that facilitates precision diagnosis and treatment of tumors [8]. Compared with invasive tissue biopsy, CTC detection has the advantages of easy sample acquisition and the ability to provide dynamic monitoring information. Compared with another liquid biopsy marker, circulating tumor DNA (ctDNA), CTC is a complete tumor cell that carries multiple omics information (genome, transcriptome, proteome, metabolome, etc.) of tumor cells [8]. Moreover, the use of live CTCs can achieve in vitro analysis of tumor cell morphology and function. CTCs counting, molecular typing, and downstream analysis have broad prospects for application in tumor efficacy evaluation, prognosis assessment, and auxiliary treatment decision-making [9].

Currently, multiple CTCs detection methods have been developed based on unique molecular substances within tumor cells as detection targets to achieve identification and classification of tumor cells [10, 11]. This method has high sensitivity and specificity and can identify individual tumor cells. However, due to the complexity of tumor, the detection efficiency of CTCs in different types of tumors and their practical guidance for the occurrence and development of tumor are issues that need to be studied further. This paper aims to explore the differences in CTC detection in different types of tumors by detecting the number and types of CTCs, and investigate the guidance value of CTC detection for the occurrence and development of different types of tumors.

## Materials and methods

### Patients

125 inpatient tumor patients were enrolled from the First Affiliated Hospital of Guangzhou University of Chinese Medicine. This study has been approved by the hospital’s ethics committee, and all participants have been informed and signed informed consent forms. All patients were confirmed through pathological results. The basic information of the enrolled patients can be found in Tables 1 and 2. Twenty healthy cancer-free individuals were enrolled as a negative control.

**Table 1:** CTCs detected in tumor patients and correlation with clinical characteristics

Features	N	CTCs counts				Epithelial CTCs				Mixed phenotypic CTCs				Mesenchymal CTCs			
		Mean	SD	t	p	Mean	SD	t	p	Mean	SD	t	p	Mean	SD	t	p
Age(years)																	
< 45	19	50.67	84.75	0.96	0.36	7.56	6.58	1	0.3	39.11	76.3	0.89	0.4	4	5.43	0.77	0.45
≥ 45	106	23.41	20.9			4.63	8.94			16.44	19.1			3.05	3.32		
Lymph node metastasis																	
With	82	30.21	37.16	1.74	0.09	5.6	10.3	1.1	0.3	21.69	34	1.42	0.16	3.89	3.63	2.94	0
Without	43	18.06	20.33			3.61	4.34			12.76	17.3			1.73	2.95		
Vascular metastasis																	
With	77	31.67	38.99	2.11	0.04	4.65	6.33	-0	0.7	23.12	35.3	1.86	0.04	3.89	3.58	2.63	0.01
Without	48	17.47	17.19			5.29	11.6			11.79	15.5			2	3.21		

Student’s t-test was performed to analyze the correlation of CTCs and clinicopathological features

**Table 2:** CTCs detected in patients with Liver cancer, Lung cancer, Cervical cancer, Nasopharyngeal carcinoma, Breast cancer, Colorectal cancer, Gastric cancer or Prostate cancer

	Liver cancer		Lung cancer		Cervical cancer	Nasopharyngeal carcinoma	Breast cancer	Colorectal cancer	Gastric cancer	Prostate cancer
No. of patients	26			29	3	6	10	17	9	5
Age(years) (Mean ± SD)	60.88 ± 10.573			64.66 ± 12.519	53.67 ± 14.189	50 ± 13.476	57 ± 13.358	58.88 ± 13.071	57 ± 17.797	63.2 ± 10.521
Sex										
Males	25			12	0	6	0	10	4	5
Females	1			17	3	0	10	7	5	0
CTC positive samples (%)	100			96.7	100	100	100	94.2	90.0	100
CTM positive Samples (%)	0			0	0	16.7	0	0	0	0
CTCs	16.5(4.75,43.25)			14(5, 29.5)		26.5(3.75, 165.25	11.5(3.75, 16)	13(3.5, 35.3)	9(6, 29.5)	29(7.5, 63)
Epithelial CTCs	73.1% 1(0,5)			81.9% 3(1, 7)	66.7	83.4% 4(0.75,13.5)	90% 2.5(1.75, 8)	71.6% 2(0, 2.5)	72% 2(0.5, 5.5)	80% 2(1, 6)
Mixed phenotypic CTCs	77% 6(0.75,38)			79.4% 8(1, 16.5)	66.7	66.7% 22(0, 142)	70% 5(0, 10.75)	82.4% 6(1, 28)	84% 7(2, 19)	80% 13(5, 53.5)
Mesenchymal CTCs		77% 2(0.75,5)		65.6% 1(0, 4)	66.7	83.4% 3(0.75, 9.75)	80% 1(0.75, 2.25)	70.6% 1(0, 2.5)	68% 2(0, 3)	80% 5(0, 9.5)

### CTCs were enriched and identified

CTCs were enriched with Canpatrol^™^ system (SurExam, Guangzhou, China). 10 mL of peripheral blood specimens were collected with EDTA-K2 anticoagulant vacuum blood collection tubes (SurExam, Guangzhou, China) from enrolled patients, and the tubes were gently inverted at least 10 times to ensure thorough mixing. The blood in the collection tubes was transferred to sample storage tubes containing red blood cell lysis buffer (154 mM NH4Cl, 10 mM KHCO3 and 0.1 mM EDTA (all from Sigma, St. Louis, USA) in deionized water), inverted and mixed 10 times, and waited for 30 min at room temperature (25 ± 5 °C) to lyse red blood cells. After the cell suspension was transferred to the filtration tube, the pump (Auto Science, Tianjin, China) valve was switched on to reach at least 0.08Mpa, the manifold vacuum plate valve was then switched on, and filtration began. Based on their larger cell volume, CTCs were captured and enriched by filtering the lysed solution through calibrated membrane filters. The counting of all CTCs in this experiment is based on the unit of per 10 mL of peripheral blood.

**Fig. 1:**
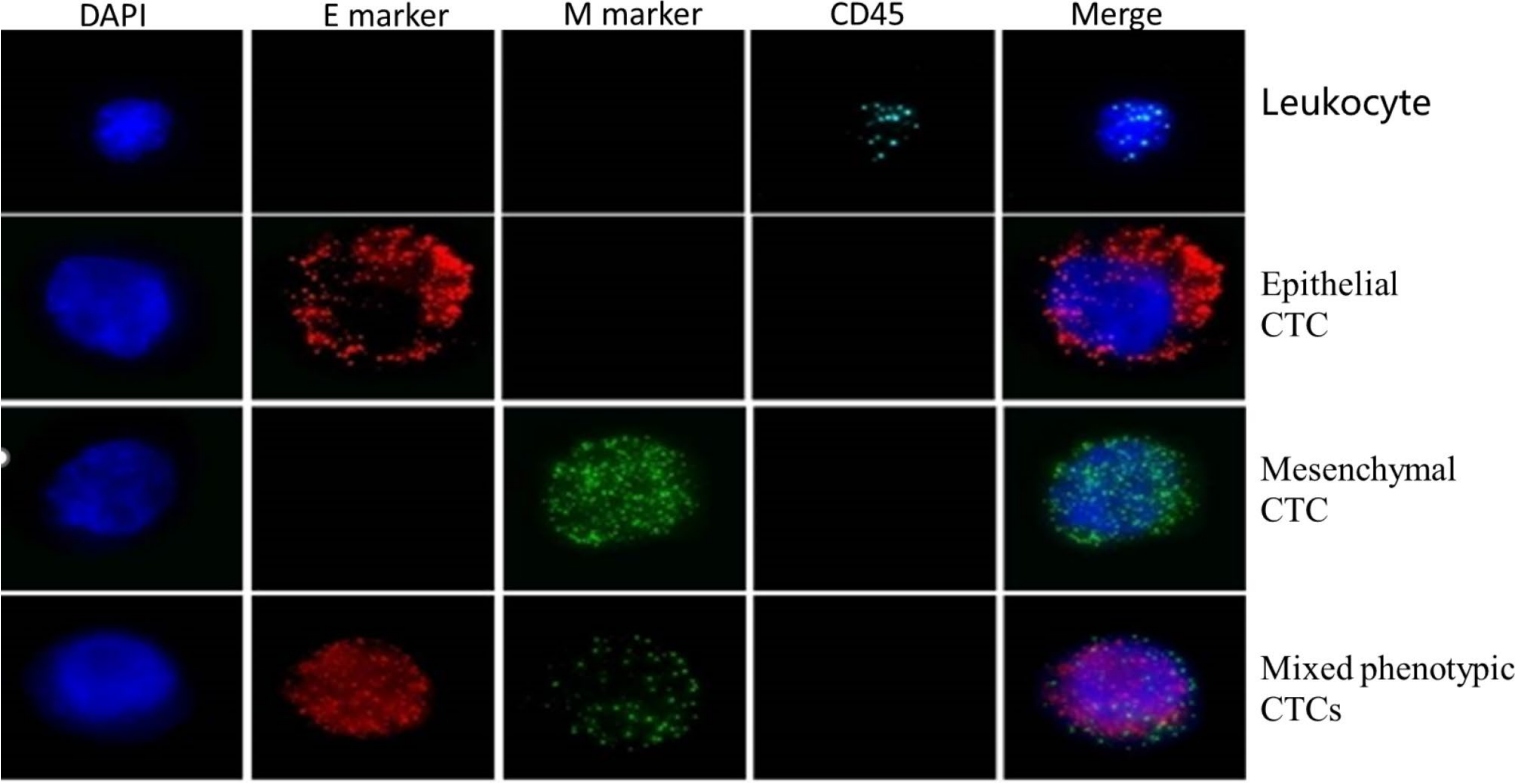
Identified EMT markers by RNA-in situ hybridization. Red fluorescence for epithelial markers EpCAM or CK8(E marker), green fluorescence for mesenchymal markers vimentin or TWIST(M marker) and white fluorescence for leukocyte marker CD45. DAPI Staining Solution is used to label nuclear (DAPI). CTC is defined as cells without CD45 which has epithelial and/or mesenchymal markers

RNA-in situ hybridization (ISH) was used for identification and characterization of the CTCs based on the branched DNA (bDNA) signal amplification technology using EMT markers. In this technique the target sequences are captured by multiple specific probes, and bDNA signal amplification probes (including the preamplifier sequence, the amplifier sequence and the label probe) conjugate with target sequences. The preamplifier sequences are designed to bind to contiguous regions on the capture probes, while other regions on the preamplifier sequences bind to multiple bDNA amplifier sequences, creating a branched structure. The label probes, which are conjugated to a fluorescent dye, are complementary to the bDNA amplifier sequences and bind to the bDNA molecule through hybridization [12]. EMT markers were labeled with different colored probes (red probes for epithelial markers EpCAM or CK8, green probes for mesenchymal markers vimentin or TWIST) to achieve CTCs subtype classification (CTCs with only red dots are epithelial CTCs, CTCs with only green dots are mesenchymal CTCs, and CTCs with both red and green dots are mixed phenotypic CTCs) (Fig. 1). In addition, leukocyte marker CD45 was labeled with white probes to prevent interference from white blood cells in the detection as previously described by the researchers [13] (Fig. 1). Samples were analyzed with a fluorescence microscope using a 40x objective (Zeiss Axio Imager.D2, Oberkochen, Germany) and automatic recognition system (SurExam MetaSystems, Guangzhou, China). CTC is defined as cells without CD45 which has epithelial and/or mesenchymal markers.

### Statistical analysis

Student’s t-test or one-way ANOVA test was performed to analyze the correlation of CTCs and clinicopathological features. The single factor analysis of variance was used to compare the CTC counts at different time points in 8 tumor patients with poor prognosis. *P < 0.05* was considered to be statistically significant. All the statistical analysis was performed with SPSS 20.0 software (IBM, USA).

## Results

### CTCs detected in tumor patients and correlation with clinical characteristics

A total of 122 cancer patients were found to have CTCs in their peripheral blood, with a positivity rate of 97.6% (122/125). CTCs could not be detected in healthy cancer-free individuals. The range of CTC counts was 1-274, with a median of 21. We further analyzed the relationship between the total number of CTCs and the number of three different subtypes of CTCs with clinical and pathological features such as age, lymph node metastasis and vascular metastasis. The results showed that there was no difference in the total number of CTCs and the number of three different subtypes of CTCs among different age groups. The total number of CTCs and the number of epithelial and mixed phenotypic CTCs were not correlated with lymph node metastasis, nor was the number of epithelial CTCs correlated with vascular metastasis. However, we found that the number of mesenchymal CTCs was significantly increased in patients with lymph node metastasis, while the total number of CTCs, the number of mixed phenotypic CTCs, and mesenchymal CTCs were all significantly increased in patients with vascular metastasis (Table 1).

### CTCs detected in patients with liver cancer, lung cancer, cervical cancer, nasopharyngeal carcinoma, et al. cancers

**Fig. 2:**
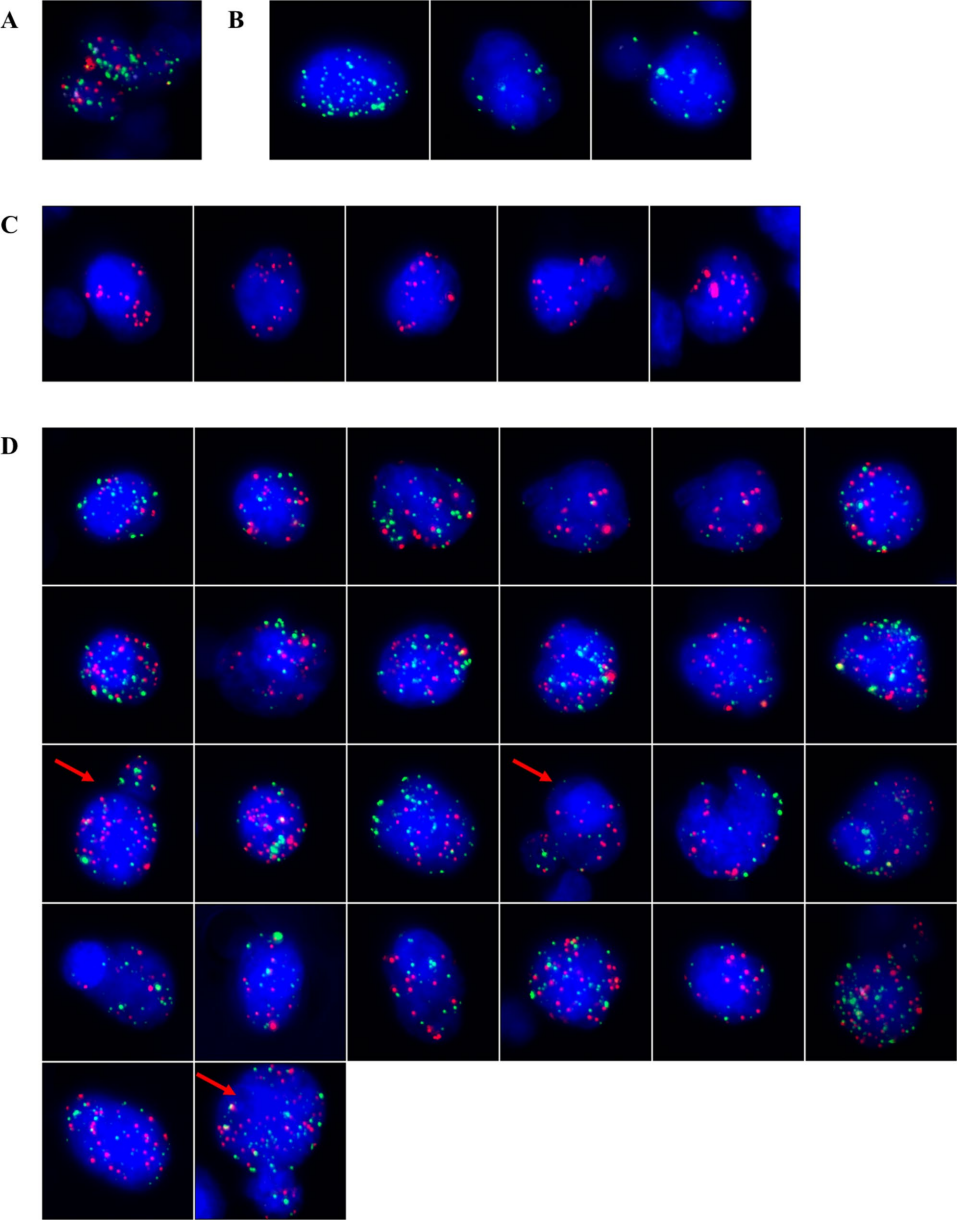
CTCs detected in a blood sample from a nasopharyngeal carcinoma patient A total of 40 CTCs were detected in this sample. 5 single migratory biophenotypic epithelial CTCs(C), 3 single migratory mesenchymal CTCs (**B**), 29 mixed phenotypic CTCs(D) containing two mixed phenotypic CTCS adhering together (→) and a tumor microembolus consisted of 2 mixed phenotypic CTCs and 1 mesenchymal CTC (**A**)were observed

We further categorized cancer patients by tumor type, including liver cancer, lung cancer, cervical cancer, nasopharyngeal carcinoma, breast cancer, colorectal cancer, gastric cancer, prostate cancer, and other groups (data not shown). We analyzed the differences in CTC counts and subtypes among different tumor types. The data showed that the detection rate of CTCs was 100% in liver cancers, cervical cancer, nasopharyngeal carcinoma, breast cancer, and prostate cancer, 96.7% in lung cancer, 94.2% in colorectal cancer, and 90% in gastric cancer. In one case of nasopharyngeal carcinoma, CTM was detected (Table 2; Fig. 2).

### The correlation between clinical staging of tumors and the detection quantity and subtypes of CTCs

**Fig. 3:**
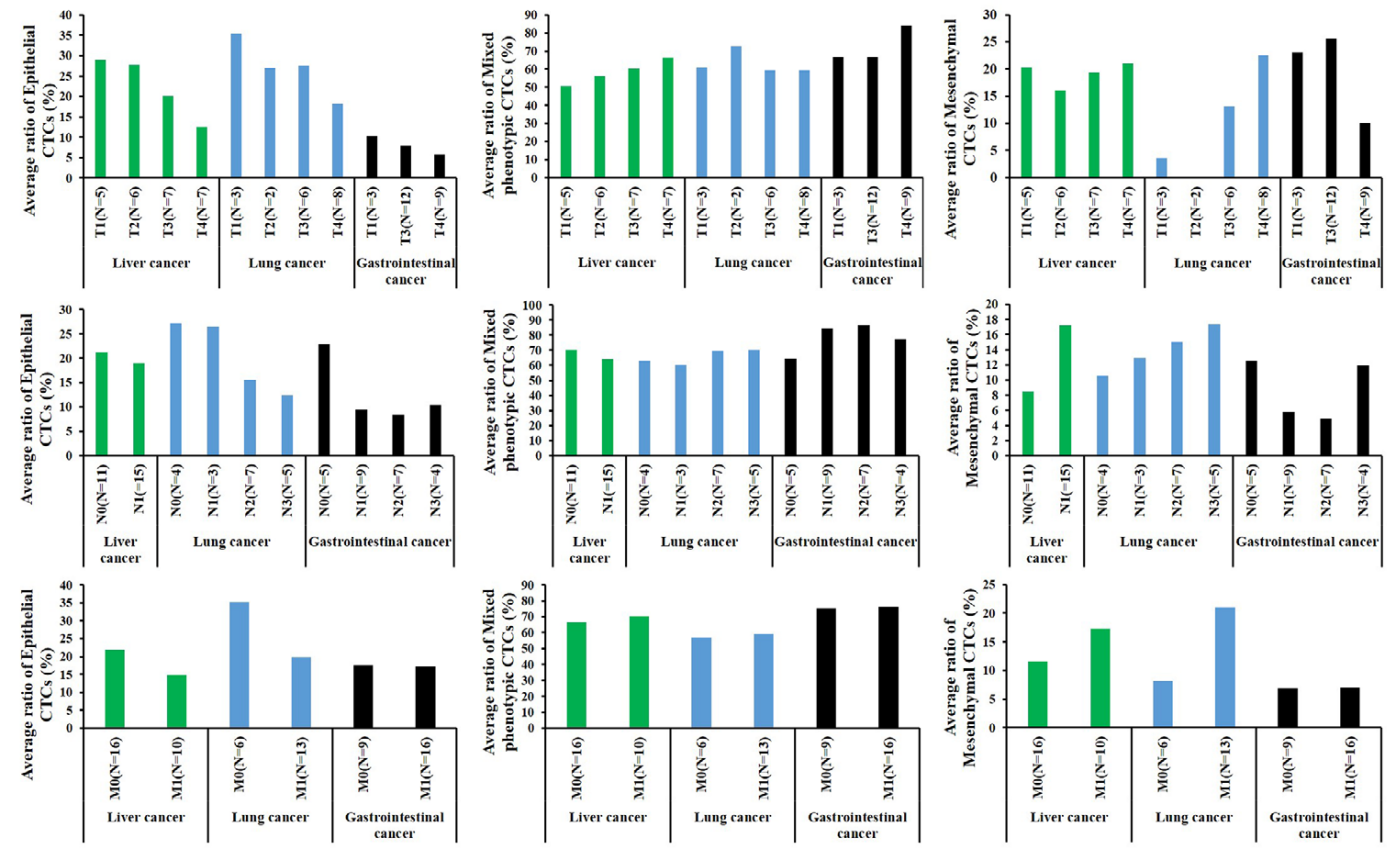
The differences in CTC detection among different clinical stages. Compared to earlier stages of cancer, the average ratio of epithelial CTCs in each positive sample decreased and the average ratio of mesenchymal CTCs in each positive sample increased in later stages of cancer. The average proportion of mixed phenotypic CTCs in later stages seem to be consistent with earlier stages

We staged different types of tumors according to the international TNM staging system and analyzed the differences in CTC detection among different clinical stages. To further analyze the changes of CTCs in different clinical stages, we separately analyzed the CTCs data based on the size and extent of the primary tumor (T), the involvement of nearby lymph nodes (N), and the presence or absence of distant metastasis (M). Our study revealed that the average proportion of epithelial CTCs decreased in later stages of liver cancer, gastric cancer, and lung cancer, compared to earlier stages. In contrast, the average proportion of mesenchymal CTCs increased in later stages of cancer, compared to earlier stages. The average proportion of mixed phenotypic CTCs in later stages seem to be consistent with earlier stages (Table 3; Fig. 3). What we need to clarify is that the results provided here are descriptive and do not involve statistical analysis.

**Table 3:** CTCs detected in different clinical stages of tumors

	Clinical staging (TNM)	Sample No.	CTCs positive sample(%)	CTCs counts (Mean)	Range of CTCs counts	Epithelial CTCs(%)	Mixed phenotypic CTCs(%)	Mesenchymal CTCs(%)
Liver cancer	T1N0M0	4	100	15	1～44	75	75	75
	T1N1M0	1	100	9	9	100	0	0
	T2N0M0	1	100	5	5	100	100	100
	T2N1M0	1	2(66.67)	24	24	2(66.67)	2(66.67)	2(66.67)
	T2N1M1	5	5(100)	26.6	1～72	4(80)	4(80)	4(80)
	T3N0M0	5	5(100)	42.6	3～93	2(40)	4(80)	4(80)
	T3N1M0	2	2(100)	13.5	5\22	1(50)	2(100)	2(100)
	T4N0M0	1	1(100)	40	40	1(100)	1(100)	0
	T4NIM0	1	1(100)	1	1	0	0	1(100)
	T4N1M1	5	5(100)	29.8	1～58	5(100)	4(80)	4(80)
Lung cancer	T1N0M0	1	1(100)	2	2	1(100)	0	0
	T1N1M0	1	1(100)	163	163	1(100)	1(100)	1(100)
	T1N2M1	1	1(100)	5	5	0(0)	1(100)	1(100)
	T1N3M1	1	1(100)	15	15	0(0)	1(100)	1(100)
	T2N0M0	2	2(100)	11	18\4	2(100)	2(100)	1(50)
	T2N2M0	2	2(100)	10.5	1\19	2(100)	1(50)	0(0)
	T3N0M0	2	2(100)	45	62\28	2(100)	2(100)	1(50)
	T3N1M0	1	1(100)	5	5	1(100)	1(100)	0(0)
	T3N1M1	3	3(100)	13.33	2～30	3(100)	1(33.33)	2(66.67)
	T3N2M1	4	4(100)	19.25	3～13	3(75)	3(75)	3(75)
	T3N3MI	1	1(100)	7	7	1(100)	1(100)	0(0)
	T4N0M1	1	1(100)	54	54	1(100)	1(100)	1(100)
	T4N1M1	1	1(100)	34	34	0(0)	1(100)	1(100)
	T4N2M1	5	5(100)	22.6	7～47	5(100)	5(100)	5(100)
	T4N3M1	3	2(66.67)	20.67	0～34	2(66.67)	2(66.67)	2(66.67)
Cervical cancer	T1N0M0	1	1(100)	6	6	1(100)	0(0)	0(0)
	T1N0M1	1	1(100)	6	6	1(100)	1(100)	1(100)
	T1N1M1	1	1(100)	33	33	0(0)	1(100)	1(100)
Nasopharyngeal carcinoma	T1N0M0	1	1(100)	129	129	1(100)	1(100)	1(100)
	T2N1M0	1	1(100)	19	19	0(0)	1(100)	1(100)
	T3N1M1	1	1(100)	3	3	1(100)	0(0)	0(0)
	T4N1M1	2	2(100)	19	4\34	2(100)	1(500)	2(100)
	T4N2M1	1	1(100)	274	274	1(100)	1(100)	1(100)
Breast cancer	T2N0M0	3	3(100)	12	5～19	3(100)	3(100)	2(66.67)
	T2N1M0	2	2(100)	14.5	14\15	2(100)	2(100)	2(100)
	T2N1M1	1	1(100)	2	2	1(100)	0(0)	1(100)
	T3N1M1	1	1(100)	3	3	1(100)	0(0)	1(100)
	T3N2M1	1	1(100)	39	39	1(100)	1(100)	1(100)
	T4N3M1	2	2(100)	7.5	4\11	1(50)	1(50)	1(50)
Colorectal cancer	T3N0M0	2	2(100)	4.5	6\3	1(50)	1(100)	1(50)
	T3N1M1	1	0(0)	0	0	0(0)	0(0)	0(0)
	T3N2M0	2	2(100)	25	37\13	1(50)	2(100)	1(50)
	T3N2M1	4	4(100)	22.75	3～34	3(75)	4(100)	3(75)
	T3N3MI	2	2(100)	21	38\4	2(100)	1(50)	2(100)
	T4N1M1	2	2(100)	49.5	61\38	1(50)	2(100)	2(100)
	T4N3M1	1	1(100)	11	11	1(100)	1(100)	1(100)
Gastric cancer	T1N0M0	1	1(100)	9	9	0(0)	1(100)	1(100)
	T3N1M1	1	1(100)	9	9	1(100)	1(100)	1(100)
	T4NIM0	1	1(100)	3	3	1(100)	0(0)	0(0)
	T4N1M1	4	4(100)	40.75	3～101	3(75)	4(100)	3(75)
Prostate cancer	T2N1M1	1	1(100)	84	84	1(100)	1(100)	1(100)
	T3N0M1	1	1(100)	13	13	0(0)	1(100)	0(0)
	T3N1M1	1	1(100)	2	2	1(100)	0(0)	0(0)
	T4N2M1	1	1(100)	29	29	1(100)	1(100)	1(100)

### CTCs detected in 8 tumor patients with poor progress

**Fig. 4:**
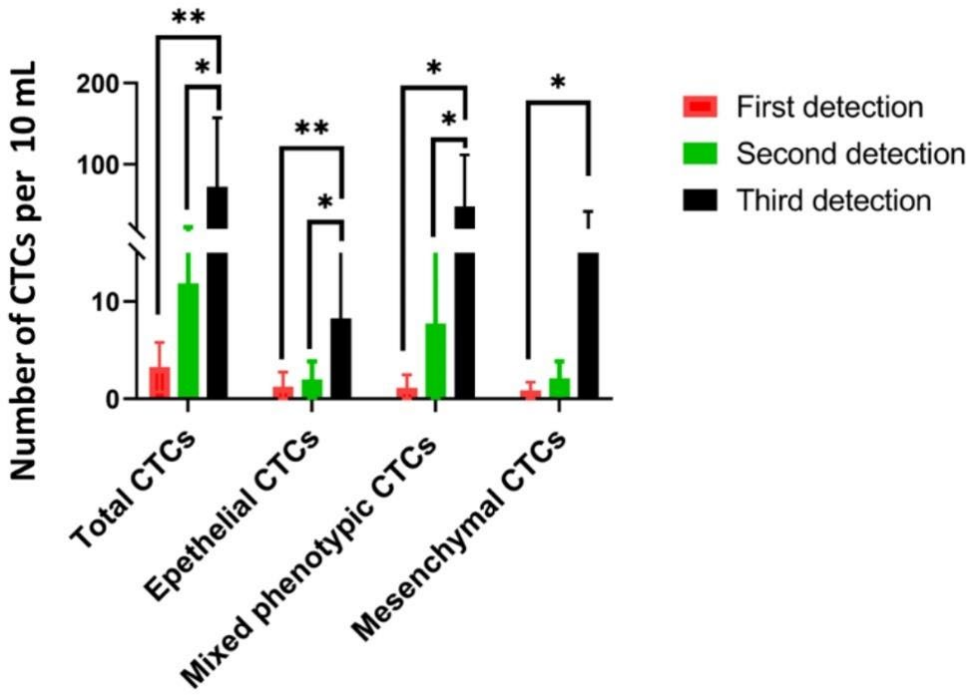
CTCs detected in 8 cancer patients with poor progress. The error bars indicate standard deviations. *P < 0.05, ** *P < 0.01*

We analyzed the changes in CTC counts and subtypes of 8 patients with poor prognosis tumors who were consecutively monitored for CTCs more than 3 times and died within one year after diagnosis. Each patient underwent CTCs detection before surgery, around 3 months after surgery, and around 6 months after surgery. The data showed that there was a significant increase in CTCs counts in the second or third detection. Especially, the number of mixed phenotypic CTCs increased significantly in the second or third detection (Fig. 4).

### CTCs detected in a blood sample from a nasopharyngeal carcinoma patient

In all enrolled cases, one nasopharyngeal carcinoma patient with a clinical stage of T4N1M1 was found to have CTM. The CTM consisted of 2 mixed phenotypic CTCs and 1 mesenchymal CTC. A total of 40 CTCs were detected in this case, including 5 epithelial CTCs (red dots), 29 mixed phenotypic CTCs (red and green dots), and 3 mesenchymal CTCs (green dots). Notably, among the 29 detected mixed phenotypic CTCs, a phenomenon of two CTCs adhering together was observed (Fig. 2).

## Discussion

Early detection and treatment of cancer greatly increase the chances of cure. Therefore, researchers have been trying to find biological tumor markers that can assist in early cancer diagnosis from various types of specimens [14–16]. However, due to the biological complexity and heterogeneity of tumorigenesis and tumors, no satisfactory tumor-specific biomarkers for early screening have been identified. Although serum tumor markers such as CEA [17], CA-199 [18], PSA [19], AFP [20], CA-125 [21], etc., which are commonly used in clinical tumor screening tests, have been widely used, none of them can accurately and specifically predict the presence or absence of tumors. EMT is a phenomenon in which epithelial cells transform into mesenchymal cells under certain physiological and pathological conditions [22, 23]. EMT mainly occurs during the process of tumor cells entering the peripheral blood circulation, characterized by the loss of epithelial cell phenotype and the acquisition of mesenchymal cell phenotype [24]. Studies suggested that EMT markers could be used for the detection or capture of CTCs. The significance and value of CTCs detection for the early diagnosis, treatment monitoring, and prognosis of tumor diseases are being gradually confirmed. Based on our results, it can be indicated a detection rate of 100% for CTCs in liver cancers, cervical cancer, nasopharyngeal carcinoma, breast cancer, and prostate cancer, 96.7% in lung cancer, 94.2% in colon cancer, and 90% in gastric cancer (Table 2). The overall detection rate of CTCs in the peripheral blood of cancer patients was 97.6% (data not shown).

The most important aspect of CTCs detection is the enrichment of CTCs, which determines the sensitivity of CTCs detection. Currently, there are several commercial systems available for CTCs enrichment, including the Canpatrol™ system, ISET system, ScreenCell system, and CellSearch system. Three systems utilize microfiltration membranes with specific pore sizes to enrich CTCs, while the CellSearch system uses a positive enrichment strategy based on the epithelial cell adhesion molecule (EpCAM) positive CTCs. In this system, EpCAM antibodies are coated on magnetic beads and specifically bind to the surface of CTCs, which are then selected under the action of an external magnetic field. The CellSearch system, based on EpCAM, is the only commercially available product approved by both the FDA (approved in 2004) and CFDA (approved in 2012) for CTCs detection in malignant tumors. Although CTCs enrichment systems based on physical properties, such as Canpatrol™, may have the disadvantages of potential contamination by other blood cells and missing certain tumor cells, their economic cost and ease of operation make them widely used methods for CTCs detection. Regarding the CellSearch system, studies have found that not all CTCs express EpCAM during the EMT process, which limits the clinical application of EpCAM-based technologies to some extent. It is important to note that each method has its own unique advantages and disadvantages, and therefore the choice of method primarily depends on our research goals and requirements. Although there are various CTCs enrichment technologies based on physical or biological characteristics, neither can effectively explain the issues of missing CTCs or leukocyte contamination, which indicates the need for further research in this area.

The detection rate of CTCs can be influenced by various factors, including tumor type, disease stage, technical sensitivity, and sample processing. In our study, we utilized the commercial product Canpatrol™ system. According to previous reports, this system has shown a detection rate for CTCs ranging from 65 to 90.18% [13, 25, 26] or even higher. In this study, to maintain the accuracy of CTCs enrichment, stringent protocols are employed during sample processing. Stringent measures are implemented to prevent membrane filtration blockage. In cases of blockage occurs, it is necessary to redraw blood samples to ensure the efficient capture of CTCs and achieve a high detection rate in cancer patients. Of course, what cannot be ignored is false positive results potentially caused by leukocytes cannot be entirely ruled out. The high detection rate of CTCs in cancer patients may provide effective assistance for early cancer diagnosis.

We conducted an analysis to investigate the association between the total number and three subtypes of CTCs in peripheral blood of cancer patients and clinical characteristics including age, lymph node metastasis, and hematogenous metastasis. Our results showed a significant increase in the number of mesenchymal CTCs in patients with lymph node metastasis compared to those without lymph node metastasis. Similarly, the total number of CTCs and the number of mixed phenotypic CTCs and mesenchymal CTCs were significantly increased in patients with hematogenous metastasis compared to those without hematogenous metastasis (Table 1). In metastatic breast cancer patients, the presence of ≥ 5 CTCs in 7.5 mL peripheral blood indicated poor prognosis [27], while in patients without colon cancer, the presence of ≥ 1 CTCs in 7.5 mL peripheral blood indicated a greater risk of recurrence and metastasis and worse prognosis. [28]. Our results also revealed that an increase in the number of CTCs or mesenchymal CTCs indicates the occurrence of tumor metastasis. Shiyang Wu, et al. study showed that mesenchymal CTCs were more common to be found in metastatic stages of cancer [13]. Our findings are consistent with previous reports indicating that mesenchymal CTCs are associated with metastasis and disease progression. [29, 30].

Studies have shown that the epithelial-to-mesenchymal transition EMT of tumor cells can promote the generation of CTCs and facilitate their survival in blood vessels, which is an important prerequisite for tumors to have high metastatic ability [31–34]. During EMT, epithelial tumor cells become more invasive and migratory, making it easier for CTCs to enter distant organs through blood vessels or lymphatic vessels. We analyzed the correlation between clinical staging (TNM) and CTCs detection. The data basically showed that the average proportion of epithelial CTCs decreased in later stages of liver cancer, gastric cancer, and lung cancer, compared to earlier stages. In contrast, the average proportion of mesenchymal CTCs increased in later stages of cancer, compared to earlier stages. The average proportion of mixed phenotypic CTCs in later stages seem to be consistent with earlier stages (Table 3; Fig. 3). Which means the counts change of epithelial CTCs and mesenchymal CTCs are more helpful for tumor monitoring. Moreover, we consecutively monitored CTC counts and subtypes more than three times in eight tumor patients with poor prognosis and observed a significant increase in CTC counts in the second or third detection. Especially, the number of mixed phenotypic CTCs increased significantly in the second or third detection (Fig. 4). These findings suggest that changes in CTC subtypes are related to the prognosis of cancer.

CTM are tumor cell clusters and are associated with high metastatic potential [7].One case of nasopharyngeal carcinoma patient was found to have CTM consisting of 2 mixed phenotypic CTCs and 1 mesenchymal CTC. Among the 26 detected mesenchymal CTCs, a phenomenon of two CTCs adhering together was observed (Fig. 2).

In conclusion, our study suggests that CTC detection can provide valuable information for tumor diagnosis and prognosis. The results indicate that different tumor types have different detection rates and subtypes of CTCs. Moreover, our findings suggest that changes in CTC counts and subtypes may be useful for monitoring disease progression and treatment response.

## Data Availability

All data generated or analyzed during this study are included in this published article. The raw data are available from the corresponding author upon reasonable request.
